# Implanted Flexible Electronics: Set Device Lifetime with Smart Nanomaterials

**DOI:** 10.3390/mi12020157

**Published:** 2021-02-05

**Authors:** Hoang-Phuong Phan

**Affiliations:** Queensland Micro and Nanotechnology Centre, Griffith University, Brisbane, QLD 4111, Australia; h.phan@griffith.edu.au

**Keywords:** flexible electronics, bioresorbable devices, long-lived implanted devices

## Abstract

Flexible electronics is one of the most attractive and anticipated markets in the internet-of-things era, covering a broad range of practical and industrial applications from displays and energy harvesting to health care devices. The mechanical flexibility, combined with high performance electronics, and integrated on a soft substrate offer unprecedented functionality for biomedical applications. This paper presents a brief snapshot on the materials of choice for niche flexible bio-implanted devices that address the requirements for both biodegradable and long-term operational streams. The paper also discusses potential future research directions in this rapidly growing field.

## 1. Introduction

Heart pacemakers, one of the first prototypes of active implanted devices, were introduced in the 1950s, not long after the invention of the first bipolar transistor [1]. Since then, the development of implanted electronic devices has been well synchronized with the advancement of microelectronics and microfabrication technologies. Today, biomedical devices have been widely deployed, saving millions of lives and significantly improving quality of life. Common applications include cochlear implants, deep brain stimulators, heart pacemakers, and defibrillators, helping to regain and regulate the functions of respective disordered organs. 

Despite the remarkable progress in bio-implanted electronics over the last 50 years since the development of heart pacemakers, current commercial and clinical devices still exhibit a significant mechanical mismatch with biotissue that limits numerous functions for multimodal implanted applications [2]. In particular, most commercial implanted active devices utilize the same engineering design concept, where the electronic components are sealed in a rigid housing made of titanium or ceramic. Long, insulated metal wires and electrodes serve as the bio-interface in these systems. This difference in mechanical properties between medical devices and soft organ poses safety risks, measurement errors, and discomfort to end-users. The use of a metal wire configuration also limits spatial physiological recording/stimulating, which is critically important to underpin complex cell bioactivities. These technical challenges trigger the need for research into the development of a new platform using flexible electronics [3,4].

The last decade has seen a spike in research into flexible electronics, initially for wearable devices, which then expanded to implantable applications, taking advantage of the matured CMOS (complementary metal-oxide semiconductor) and MEMS (microelectromechanical systems) technologies [5]. These new platforms provide ideal integrations with the soft, curvilinear, and elastic tissues and the unique capability of multimodal functions, enabling large surface electrophysiological mapping. These ground-breaking features open unprecedented opportunities for bioelectronic interfaces tapping into intracellular and extracellular pathways and offering powerful probes for the diagnosis and treatment of neurological disorders, cardiac diseases, and many other complex health issues [6].

In general, the lifespan of flexible implanted device can be categorized into two main streams, biodegradable and long-lived systems. This subdivision is relatively analogous to traditional implanted mechanical components, where some devices need to safely dissolve after a certain period of time (e.g., absorbable stitch, temporary skeletal anchorage), while others must permanently remain inside the human body (e.g., protheses for joint and hip replacement). For flexible electronics, the construction materials play a decisive role in defining the operational timescale of the corresponding implanted devices.

## 2. Materials for Biodegradable Implanted Devices

In several cases, implanted devices should gradually dissolve in biofluids once they have provided the targeted functions and are no longer needed. The self-degradation in biofluids offers significant advantages as it eliminates the requirement for extra surgeries or operations to replace or remove the previously implanted devices [7]. This property not only reduces the cost of medical treatment but also minimizes the risks associated with additional operations to patients. The self-degradation (or transience) of flexible implanted devices is realized through the use of relevant materials for all constituent components, from semiconductors and dielectric layers to metal interconnects and host substrates that exhibit significant hydrolysis reaction, a chemical process that gradually dissolves materials through the reaction with water.

**Figure 1:**
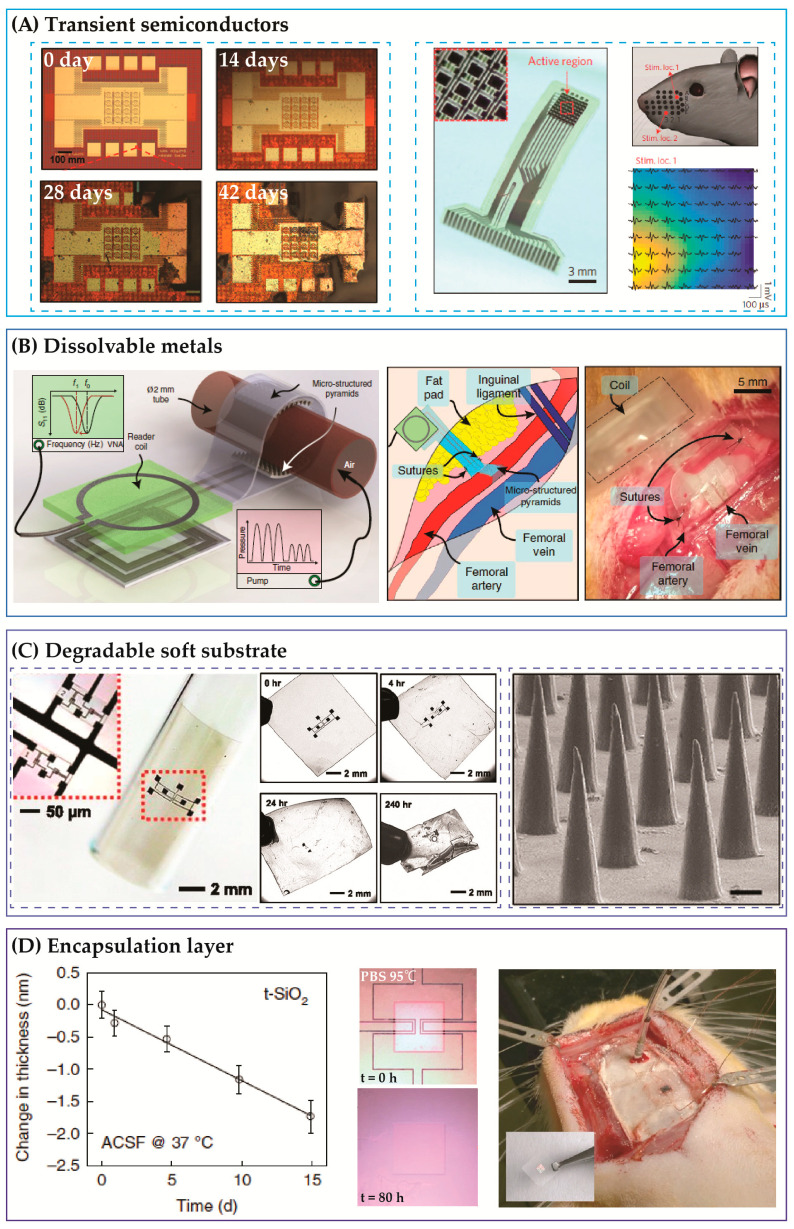
Material systems for biodegradable electronics require all components of encapsulations, metals, semiconductors, and substrates exhibiting the dissolvable property. (**A**) Silicon nanomembrane as functional semiconducting element. Left: Transient Si based complementary metal-oxide semiconductor (CMOS) devices [9]. Right: An array of Si thin film transistor (TFT) for neuron recording [12]. (**B**) Example of dissolvable metals, where Mg was utilized to form antenna for wireless stimulator and sensors [15]. (**C**) Poly(lactic-co-glycolic acid) (PLGA) as a biodegradable substrate. Left: Transient electronics on a PLGA substrate [16]. Right: Examples of PLGA films used for drug delivery [17]. (**D**) Temporary encapsulation layer capable of dissolving in biofluids. Left: Hydrolysis rate of silicon oxide. Right: An intracranial pressure sensor encapsulated with silicon oxide (SiOx) [18].

Silicon is among the most common choices of bioresorbable semiconductors (Figure 1a) [8,9]. Low-doped silicon nanomembranes (e.g., phosphorous doped, 10^17^ cm^−3^ concentration) dissolve in water to form silicic acid (Si + 4H_2_O → H_4_SiO_4_ + 2H_2_) at a rate of approximately 3 nm/days at 37 °C in 0.1M PBS, pH 7.4 [9,10]. The data indicate that a 100 nm thick Si-based thin film transistor (TFT) can gradually disappear from the human body after a month of implantation. Interestingly, the dissolution rate of Si significantly depends on the carrier concentration, where highly doped nanomembranes (10^20^ cm^−3^) can last for a much longer time that intrinsic or low doped materials. In addition, the dissolution rate also correlates to pH levels as well as the contents of surrounding biofluids. For instance, increasing pH levels (e.g., from 7.4 to 12) could markedly accelerate the hydrolysis reaction of Si [11]. These features suggest promising approaches to actively control the operating lifetime of Si-based electronics through engineering processes (e.g., controlling carrier concentrations) or biomedical methods (using medicines as catalysts for the dissolution reaction). An array of 300 nm thick Si nanomembranes spatially distributed on a two-dimensional mesh has been successfully demonstrated to detect normal physiology activities, both in acute and chronic recordings in mouse models [12]. The device dissolved in PBS, pH 10 after 15 days, proving the potential of transient electronics for monitoring and treatment of neuron activities. Besides Si, Ge and ZnO have also been proven to be bioresorbable, exhibiting relatively faster dissolution rates that can be employed to construct temporary implanted soft electronics [13,14].

Along with semiconductors, the selection of metals that offer functional bio-interfaces and interconnects, and at the same time facilitate the dissolvable process, is critically important. In this regard, Mg, Mo, Zn, and W have been widely employed due to their significant hydrolysis rate when in direct contact with water [19,20]. Even at room temperature, Mg exhibits a high dissolution rate of 1.68 µm/day [21]. Examples of bioresorbable devices using Mg as interconnects and functional elements include the wireless arterial-pulse sensors for blood flow monitoring post-vessel surgery [15] (Figure 1b). The device employs a pair of multiple-loop Mg coils to wirelessly transfer electrical signals through skin. The blood flow leads to a deformation in the blood vessel, which changes the capacitance between the two Mg coils. This change in capacitance can be wirelessly detected based on a shift in the inductive coupled frequency, read by an external coil antenna placed on the surface of skin. The Mg sensors were found to biodegrade after 12 weeks of implantation in mice. Another example of Mg-based flexible electronics is a sciatic nerve stimulator capable of delivering electrical stimulating signals using an inductive coupling method. The whole device completely dissolved in PBS at 37 °C after twenty-five days [22].

Another imperative component for bioresorbable devices is the hosting soft substrate. In this regard, poly(lactic-co-glycolic acid) (PLGA) films possess ideal properties due to their biocompatibility and degradation that provide toxicologically safe byproducts into biofluids [16]. Experimental studies on the in vitro dissolution of thin PLGA films (5 µm to 10 µm thick) show a heterogeneous bulk degradation process in 0.2M PBS at 37 °C, with approximately 35% mass loss after six weeks (for a copolymer ratio of 75:25) [23]. In addition, changing the copolymer ratio from 75:25 to 50:50 increases the degradation rate in the films. The dissolution rate of PLGA also depends on the film thickness, temperatures, and the pH levels of biofluids. Another advantage of PLGA for implantable applications is the versatility in its fabrication processes—such as solvent casting, compress modelling, and extrusion—that ease the integration of microelectronics onto a soft substrate [17,24]. These features make PLGA a fascinating material for not only the host substrate for biodegradable electronics but also for constructing components for drug delivery and cell growth scaffold [25] (Figure 1c). Along with PLGA, polymers such as silk and polyvinyl alcohol as well as natural wax have also proven their high feasibility to host transient implanted electronics [26,27].

Prior to dissolving in fluidic environments, flexible devices should maintain a stable performance until the dissolution process is triggered. This is controlled using a thin encapsulation layer that can protect the integrated devices in a certain period of time, depending on the requirement. Silicon oxide (SiOx) has been widely used as a temporary encapsulation layer for biodegradable devices, due to its lower dissolution rate (e.g., less than 0.1 nm/day in PBS pH 7.4 at 37 °C) compared to Si and Mg [28,29] (Figure 1d). Along with SiOx, SiN and MgO are alternative choices, which possess faster dissolution rates that can allow for a more rapid device degradation, for instance after a few days or few weeks of implantation [18,30]. The use of encapsulation layers can help maintain proper functions of integrated electronics components such as TFT and temperature sensors. Once these barrier layers are partly or completely removed, the direct contact between water and underlying functional elements will facilitate the degradation of the whole device. However, for some mechanical sensing devices (pressure sensors, strain sensors), the change in the devices thickness (due to the gradual dissolution of the encapsulation layer and substrate) leads to a drift in the readout signals. A promising solution for this problem is using smart mechanical architecture that maintains sensor performance while undergoing the dissolution process. As such, by balancing the position of the mechanical neutral axis and the bending stiffness, Yang et al. demonstrated an intracranial pressure sensor with reproduceable output voltages even when the encapsulated membranes are degraded [31]. The key concept of this design is that the multiple encapsulation layers consist of Si nanomembranes and PLGA. The sensitivity of the pressure sensor is proportional to the ratio between the distance from the sensing element to the neutral axis and the bending stiffness of the Si/PLGA bilayer. This sensitivity can be maintained at a constant value under the dissolution process by using an optimal ratio between the initial thickness of the Si nanomembrane and the PLGA film.

So far, the lifetime of the biodegradable devices mainly depends on the timescale of the encapsulation layers, which is fundamentally designed based on their hydrolysis rates, thickness, and crystallinity. This passive approach requires predetermination of the operational lifetime and cannot be subsequently modified regardless of the treatment outcomes and conditions. Recent studies suggest new active approaches where the degradation process can be triggered on-demand rather than relying on the predefined parameters [32,33]. Thermal-triggered transience has emerged as an innovative method to activate the dissolution reaction. Park et al. employed wax containing microdroplets of methane sulfonic acid (MSA) as a triggerable encapsulation [34]. Under thermal stimuli, the coating wax is melted, which releases the encapsulated acid droplets. These acid droplets serve as catalyst agents to chemically dissolve the underlying electronic components, leaving biocompatible byproducts into biofluids. By choosing a relevant mixing ratio of MSA and wax, degradation can occur at relatively low temperatures (e.g., 45 °C), which are considered safe to users. A combination of wireless communication (e.g., inductive resonant coupling) enables remote trigger of the self-degradation once the implanted devices are no longer needed.

## 3. Materials for Long-Lived Implantable Devices

Treatment of several chronic diseases requires electronics to stay for several decades inside the body of patients without replacement [35]. Representative examples for these applications are pacemakers and defibrillators that should continuously provide heart regulation functions over the lifetime of the patients. As a titanium or ceramic housing is no longer applicable to the soft platform, enabling mechanical flexibility, maintaining a sufficiently long operational timescale (e.g., several decades) is a great challenge. There are three key components to maintain a sustainable operation for flexible implanted electronics, which are (i) a robust polymeric substrate to host electronic components, (ii) a long-term encapsulation layer for integrated circuits, and (iii) a stable Faradaic interface for recording and stimulation.

**Figure 2:**
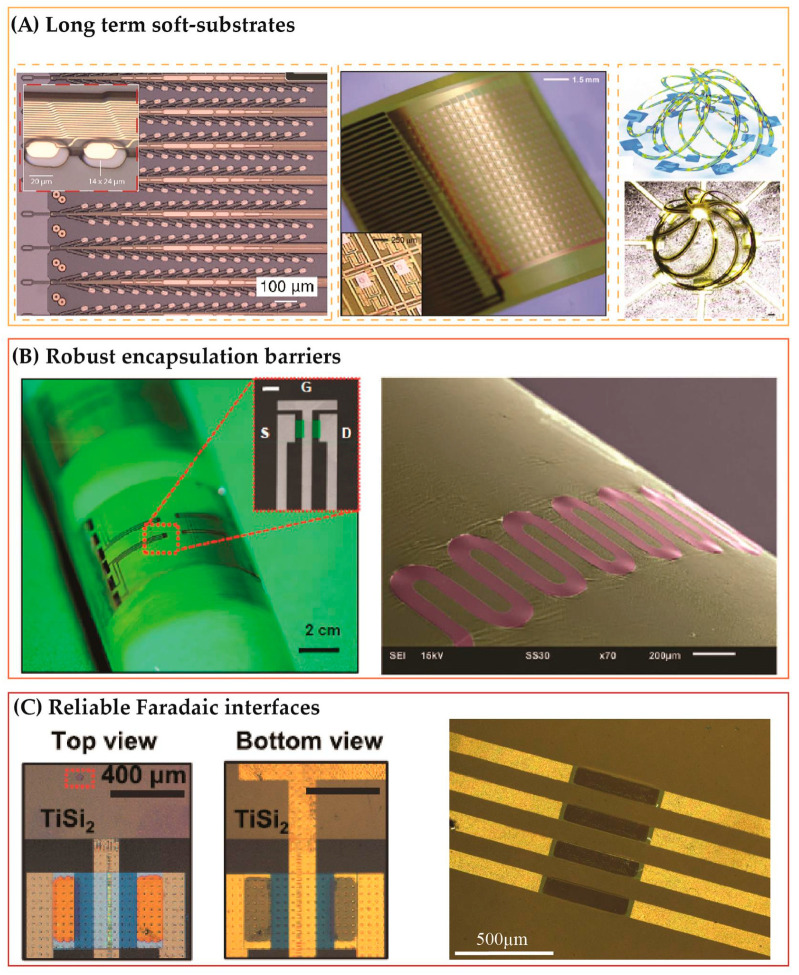
Long-lived electronics are constructed by materials that can tolerate to biofluidic corrosion and ion diffusion. (**A**) Stable soft substrate using polyimide. From left to right: Photograph of tree-probe configuration, [37] array of TFTs on a bendable polyimide film, [39] 3D polyimide structures as a scaffold for cell growth [40]. (**B**) Bio-barriers. Left: tri-layer of parylene-C/HfO_2_/SiO_2_ [41]. Right: A stretchable SiC membrane [42]. (**C**) Faradaic interfaces for recording and stimulation. Left: Si/TiS_2_ electrodes [43]. Right: Highly doped transferred SiC resistors [44].

Polyimide is a common soft substrate used to layout other electronic devices onto due to its chemical inertness and thermal stability. Polyimide, in some cases known as Kapton films, has been used as a flexible insulator and a protective layer in extreme environments such as high temperatures and increased corrosion [36]. Polyimide as the substrates for implanted electronics have been used in soft tree-probe electrodes for deep neural stimulation (Neuralink) [37], a micromesh platform consisting of arrays of microelectrodes [38], and a bendable substrate for a high density of integrated TFTs [39] (Figure 2a). The material is highly compatible with standard microfabrication processes, which allows lithography, wet etching, dry etching, and laser machining to form multiple microarchitectures. This advantage enables the development of soft two-dimensional (2D) structures such as serpentines, springs, and spiral shapes out of polyimide films, offering excellent flexibility and stretchability to soft electronics. Utilization of strain engineering (e.g., pre-strained method and residual strain) to polyimide transforms 2D structures into 3D configurations that provide new biological functions, including scaffolds for neuron growth and 3D electrophysiological mapping [40].

Polymer substrates serve as long-term anchors for electronics to stay on. However, polymers solely cannot prevent the diffusion of water molecules into underlying electronics due to the presence of pinholes in these materials. Therefore, it is imperative to have a thin encapsulation layer to protect integrated devices from water and ions. While SiOx exhibits hydrolysis reaction, a sufficiently thick SiOx layer (e.g., 1 µm) could significantly slow down the dissolution of other integrated electronic components (Figure 2b). A 1 µm thick SiOx film was employed as the encapsulation layer for long-lived Si TFT electronics, with an estimated operational lifetime of approximately seventy years at 37 °C [45,46]. However, a thick SiO_x_ layer could reduce the mechanical flexibility of devices. In addition, the significant ion diffusion rate into oxide could affect the stability and performance of the underlying electronics. This is a critical issue for bio-integrated electronics as biofluids contain numerous types of alkali metal ions such as K^+^, Na^+^, and Ca^2+^. The diffusion of these ions through a barrier layer has been reported to accelerate the failure of underlying components (e.g., shifts in the threshold voltage of TFT). A combination of polymer films CVD (Chemical Vapor Deposition) grown on thin oxide films (e.g., parylene-C/HfO_2_/SiO_x_ with thicknesses of 50 nm/50 nm/100 nm, respectively) can enhance mechanical flexibility as well as reduce the ion diffusivity into the barrier [41]. The implementation of the tri-layer structure also shows a slow hydrolysis rate, thereby improving the longevity of devices. However, the requirement for different synthesis methods and micromachining techniques for each staking layer could be a drawback toward a simple integration of semiconductor components onto soft platforms. Using a multiple-layer barrier could also diminish capacitive coupling, which results in a diminution in the signal-to-noise ratio. Recent studies suggest that SiC, a wide band gap material, can outperform silicon oxide in both water impermeability and ion barrier. A 230 nm thick SiC crystalline film can last for over 60 days at 96 °C (0.1M PBS, pH 7.4) without significant change in the thickness [44]. The result indicates the SiC films can last over 100 years at human body temperature. Two-dimensional SiC membranes with complex architectures such as serpentines and spiral shapes have been successfully transfer-printed onto polyimide, indicating the promising possibility for long-lived barrier layers capable of bending and stretching [42].

Nonconductive encapsulation layers help protect integrated components; however, for stimulation and some recording applications, a direct interface between active electronics and biotissue is required. In this regard, highly conductive metals such as Ti and Au have been widely used in implanted devices [47]. Nevertheless, as water molecules can diffuse through pinholes presenting in these metals, materials that possess good electrical conductivity along with excellent ion/water barrier are highly desired. Li et al. demonstrated the use of TiSi2 as a long-term Faradaic interface that can last for several decades under human body conditions [43] (Figure 2c). A disadvantage of metal interfaces is their limited functions, which hinder the tailoring of optogenetic stimulations [48]. In this regard, their excellent chemical inertness and long-term stability suggest that wide band gap materials (e.g., SiC and GaN) are promising candidates for robust and multifunctional semiconductor and biotissue interfaces [49,50,51,52,53,54].

## 4. Future Directions

### 4.1. Fundamental Research

Flexible implanted electronics have attracted significant research attention over the last decade, and both biodegradable and long-lived implanted electronics are expected to play an important role in future modern biomedical devices. Active research directions in this field in the coming years include validation of the efficacy of these implanted systems, safety and reliability issues, wireless communication and data processing, and strategies for active control of the device lifetime.

For biodegradable devices, as the whole systems gradually dissolve into the body, further investigation is required to elucidate the cytotoxicity of the dissolved materials. Understanding and quantifying the tolerable upper intake levels of all degraded elements are critical to ensuring safety. In addition, before reaching the point of degradation, it is imperative to maintain a consistent functional performance. In this regard, active approaches that can trigger the destructive process on-demand show promising possibilities. Development of innovative and simple techniques capable of wirelessly or remotely controlling the degradation process in implanted devices upon the accomplishment of the treatment is expected to be an exciting research direction. Furthermore, so far, most of the developed devices only exhibit a partly dissolvable performance. Some constituent components such as batteries and microprocessors (e.g., programable integrated circuits, and wireless communication units) are deployed from commercially available products, which are not bioresorbable. Therefore, there is a need to advance the development of microprocessors on soft platforms (i.e., soft CMOS technology) toward fully biodegradable implanted electronics.

For long-lived flexible applications, the device lifetimes are mainly estimated in a simulated biofluidic environment. The properties of the electronics/biotissue interface over a long term are still not fully understood. Therefore, further in vitro and in vivo work is required to investigate and maintain the stability of the bioelectronic interface. Another critical issue for long-lived implantable devices is the power source. Currently available batteries for biomedical applications are too bulky and generally last less than a decade. A possible solution is wireless power and signal transfer through near field communications (NFC) or radio frequency identification (RFID). It is of significant interest to develop wireless communication components with smart materials and architectures that can offer sustainable performance. Furthermore, relatively thick oxide films are mainly used as the barrier protecting electronics from corrosive biofluids as well as preventing current leakage. The design for this encapsulation layer must be compromised, as a thicker layer would enhance device lifetime but at the same time reduce the mechanical flexibility and sensitivity of the functional device. A potential approach to this technical problem is the development and implementation of high-quality nanothin materials that exhibit extremely low hydrolysis and ion diffusion. Among these, wide band gap materials such as silicon carbide, boron nitride, and gallium nitride are promising candidates.

Biotissue, by its nature, exhibits significant mechanical elongation and deformation. Incorporating these intrinsic properties into soft electronics to facilitate a seamless integration of devices and biological organs will be of significant interest. Current technologies for degradable and long-lived systems still exhibit numerous design constraints, such as the requirement for bio-barrier layers fully encapsulating underlying electronics and the complexity of multiple inorganic and organic layers. The development of innovative micro/nanoarchitectures that can overcome the extreme mechanics inherited from biological tissue is a hot topic of future research.

### 4.2. Pathway toward Real-World Applications

Several technologies presented in this perspective have immediate impact on fundamental medical research (e.g., NeuroLux Optogenetics Systems, Neuralink neural electrodes). However, these devices have not yet been translated into practical uses. Translation of fundamental biomedical discoveries into real-world applications requires systematic clinical trials. To date, flexible devices have been demonstrated on cultured cells or animal models for proof of concept. In many cases, animal tissues represent different properties and behaviors from those of humans. Therefore, further clinical investigations are the key to revealing the insight of (i) the efficacy of flexible electronics on health monitoring and treatment in humans, (ii) the potential of risk and side effects, and (iii) the stability of the bio- and electronics interfaces over long-term implantation. There was significant concern regarding the reliability and efficacy of pacemakers when they were first introduced over six decades ago. Today, these devices have marked their milestone in the history of human medical technologies. Implanted flexible electronics must pass along the same pathway toward clinical practice. Significant research interest from the scientific community and extensive investment from the MedTech sector make this translational process a promising possibility.

During the current global crisis, the COVID-19 pandemic, the potential of personal health care devices has become increasingly apparent [55]. Implanted flexible electronics with their multiple functions could offer an effective approach for real-time monitoring and early detection of infection, as well as symptom development. The information of body temperatures, heart rates, and respiration patterns obtained from implanted devices can serve as useful indicators and guidelines for diagnosis and subsequent actions [56,57]. Furthermore, recent reports suggested that, due to the requirement for social distancing during the COVID-19 pandemic, monitoring of implanted devices in patients with chronic diseases (e.g., cardiac implantable electronics) has been partly transformed from conventional clinical visits to remote services. This change in clinical activities brings new opportunities and benefits to both health service receivers and providers [58]. Examples of these benefits include early detection of actionable events, reduction in hospital visits, and enhanced treatment outcomes. The development of implantable flexible electronics well fits into this new trend in remote and personalized health care services. In the era of the internet-of-things and big data, healthcare decisionmakers can assess the physiological status of patients in a more timely and cost-effective manner with the help of implanted devices that are wirelessly connected to “the Cloud”. To make this perspective a reality, there is an increasing demand for intense multidisciplinary collaboration to address the unmet technical and clinical challenges in flexible electronics.

## Data Availability

Not applicable.
